# National mitigation potential from natural climate solutions in the tropics

**DOI:** 10.1098/rstb.2019.0126

**Published:** 2020-01-27

**Authors:** Bronson W. Griscom, Jonah Busch, Susan C. Cook-Patton, Peter W. Ellis, Jason Funk, Sara M. Leavitt, Guy Lomax, Will R. Turner, Melissa Chapman, Jens Engelmann, Noel P. Gurwick, Emily Landis, Deborah Lawrence, Yadvinder Malhi, Lisa Schindler Murray, Diego Navarrete, Stephanie Roe, Sabrina Scull, Pete Smith, Charlotte Streck, Wayne S. Walker, Thomas Worthington

**Affiliations:** 1 Conservation International, 2011 Crystal Drive #600, Arlington, VA 22202, USA; 2 Earth Innovation Institute, 98 Battery Street, Suite 250, San Francisco, CA 94111, USA; 3 The Nature Conservancy, 4245 Fairfax Avenue, Suite 100, Arlington, VA 22203-1606, USA; 4 Land Use and Climate Knowledge Initiative, Global Philanthropy Partnership, 2440 N Lakeview #15A, Chicago, IL 60614, USA; 5 College of Engineering, Mathematics, and Physical Sciences, University of Exeter, Exeter EX4 4QF, UK; 6 Department of Environmental Science, Policy, and Management, University of California, Berkeley, CA 94720, USA; 7 Department of Agricultural and Applied Economics, University of Wisconsin-Madison, Madison, WI 53706, USA; 8 United States Agency for International Development, 1300 Pennsylvania Avenue NW, Washington, DC 20004, USA; 9 Department of Environmental Sciences, University of Virginia, Charlottesville, VA 22903, USA; 10 Environmental Change Institute, School of Geography and the Environment, University of Oxford, Oxford OX1 3QY, UK; 11 The Nature Conservancy, Calle 67 #7–94, Piso 3, Bogota, Colombia; 12 Earth Day Network, 1616 P Street NW, Suite 340, Washington, DC 20036, USA; 13 Institute of Biological and Environmental Sciences, University of Aberdeen, Aberdeen AB24 3UU, UK; 14 Department of International Politics, University of Potsdam, D-14469 Potsdam or Climate Focus, Schwedter Strasse 253, 10199 Berlin, Germany; 15 Woods Hole Research Center, Falmouth, MA 02540, USA; 16 Department of Zoology, University of Cambridge, Cambridge CB2 3QZ, UK

**Keywords:** natural climate solutions, climate mitigation, protection, land management, restoration, Paris Agreement

## Abstract

Better land stewardship is needed to achieve the Paris Agreement's temperature goal, particularly in the tropics, where greenhouse gas emissions from the destruction of ecosystems are largest, and where the potential for additional land carbon storage is greatest. As countries enhance their nationally determined contributions (NDCs) to the Paris Agreement, confusion persists about the potential contribution of better land stewardship to meeting the Agreement's goal to hold global warming below 2°C. We assess cost-effective tropical country-level potential of natural climate solutions (NCS)—protection, improved management and restoration of ecosystems—to deliver climate mitigation linked with sustainable development goals (SDGs). We identify groups of countries with distinctive NCS portfolios, and we explore factors (governance, financial capacity) influencing the feasibility of unlocking national NCS potential. Cost-effective tropical NCS offers globally significant climate mitigation in the coming decades (6.56 Pg CO_2_e yr^−1^ at less than 100 US$ per Mg CO_2_e). In half of the tropical countries, cost-effective NCS could mitigate over half of national emissions. In more than a quarter of tropical countries, cost-effective NCS potential is greater than national emissions. We identify countries where, with international financing and political will, NCS can cost-effectively deliver the majority of enhanced NDCs while transforming national economies and contributing to SDGs.

This article is part of the theme issue ‘Climate change and ecosystems: threats, opportunities and solutions’.

## 1. Introduction

Achieving the goal of the 2015 Paris Agreement on climate change^1^ to limit warming well below 2°C requires rapid decarbonization of all economic sectors, in parallel with a scale-up of carbon sequestration [1,2]. Immediate actions are necessary in both energy and industry, as well as in the agriculture, forestry and land use (AFOLU) sectors [3–5]. Nationally determined contributions (NDCs) are domestically determined and define national climate goals and action plans, first submitted in 2014 and 2015, and are essential to the Paris Agreement. Parties to the Paris Agreement are expected to implement their NDCs after 2020 when the agreement enters the implementation phase. Parties are also strongly encouraged to submit new or revised NDCs reflecting enhanced ambition by 2020 before the Paris Agreement succeeds the Kyoto Protocol.

The Agriculture, Forestry, and Other Land Use (AFOLU) sector is included in many countries' first NDC, but with differing levels of specificity [6]. A study published in 2017 synthesized and clarified the potential activities, scale and geographies for AFOLU sector climate mitigation, demonstrating that natural climate solutions (NCS) can reduce and reverse AFOLU sector emissions and provide about a third of the climate mitigation needed by 2030 to meet the goals of the Paris Agreement [7]. NCS are a suite of protection, restoration and improved land management pathways that generate climate change mitigation outcomes. Each NCS pathway is a discrete and quantifiable type of action to avoid greenhouse gas (GHG) emissions and/or increase carbon sequestration in forest, savannah, agricultural lands or wetlands. NCS can also be referred to as nature-based solutions (NbS), although this is a broader term that also refers to climate adaptation, food security, water security, human health, and social and economic development derived from nature [8].

To facilitate more specific and enhanced NDCs, and their implementation, here we update the 2017 NCS study [7] with a synthesis and country-level disaggregation of the most recently published datasets [9–11], and with new datasets released here, on biophysical and cost-constrained NCS potential. We constrain this analysis to 12 NCS pathways that represent the bulk of NCS potential (86%) and are distinguished by their ability to deliver ecosystem services [7] which are linked to a range of sustainable development goals (SDGs) [12]. We also limit this study to tropical countries, which merit specific investigation from a climate perspective because they harbour the majority of global NCS potential (61%, [7]), have the highest rates of forest loss and gain [13,14], and the highest gross carbon fluxes [15,16] compared with temperate and boreal latitudes.

Nevertheless, large opportunities also exist for NCS to mitigate GHG emissions in non-tropical countries [7], and future work is important to extend analyses to temperate and boreal regions. Improving our estimates of cost-constrained NCS potential for countries outside the tropics faces additional data availability and accounting challenges, such as the scarcity of cost data for reforestation [9], and greater challenges in distinguishing forest conversion from rotational forestry or natural disturbance [14].

NCS will be essential for enhancing NDC ambition in many countries, as required to balance anthropogenic emissions with removals by mid-century and achieve the temperature goal of the Paris Agreement (article 4.1 of the Paris Agreement). We, therefore, evaluate the potential contribution of cost-effective NCS to balance anthropogenic emissions in each country.

To better understand not only the magnitude of NCS, but also where and how to advance NCS deployment, we also analyse factors influencing the feasibility of national implementation (governance, financial capacity), and identify groups of countries that share similar portfolios of their largest NCS pathways. Our research draws from methods used in a recent study that synthesized analyses of national terrestrial mitigation potential with financial and governance constraints and other variables to prioritize geographies and actions needed to deliver on the Paris Agreement [17].

Prior studies have classified groups of countries with respect to the need for protection and restoration of forests to inform the structure of international climate financing and policy mechanisms [18,19]. Here, we expand on these studies to develop a classification of NCS country groups that includes savannahs, agricultural lands and wetlands in addition to forests, and to include improved management of working lands in addition to protection and restoration of native ecosystems.

## 2. Material and methods

### (a) Selection and classification of pathways

We analysed the pathways that provide at least three of the four additional benefits described by Griscom *et al*. [7]: biodiversity (habitat), air (filtration), water (filtration and/or flood control) and soil (enrichment). Among these, we excluded avoided grassland conversion, which had the smallest mitigation potential and no available globally spatial grassland conversion dataset that would allow for country-level disaggregation. For the same reason, we were unable to include seagrass in our coastal wetland pathways. We classified the remaining 12 pathways into one of three types of activity (‘protect,’ ‘restore,’ ‘manage’) and three cover types (forest/savannah, agriculture, wetland), resulting in six categories (combinations of ‘pathway type’ and ‘cover type’ in table 1). None of the 12 pathways fell into the remaining three of the nine possible categories (protect agriculture, restore agriculture, manage wetlands).

**Table 1.** Methods summary for 12 NCS pathways organized by biome and pathway type. See the electronic supplementary material for details of methods.

pathway type	cover type	pathway	methods overview and pathway definition
protect	forest	avoided forest conversion	generated new disaggregation to the country level of global biophysical and cost-constrained potential from Griscom *et al.* [7], who derived avoided CO_2_ from [20]; corrections made using [21]; ‘forest’ defined as > *ca* 30% tree cover; excludes loss of ‘managed forest’ except for the inclusion of emissions due to conversion to subsistence agriculture; forested peatlands and mangroves excluded to avoid double counting; determined as avoiding emissions from baseline forest conversion rate (2000–2012)
protect	wetland	avoided peat impacts	used country-level biophysical potential and applied cost constraint from [7]; includes avoided emissions of above- and belowground biomass and soil carbon due to avoided degradation and/or loss of freshwater wetlands
protect	wetland	avoided mangrove loss	generated new pantropical estimates of national emissions (and avoidable emissions) from biomass and soil organic carbon resulting from mean annual mangrove loss from 1996 to 2016; mangrove loss rate was derived from [22]; mangrove biomass was derived from [23], and soil organic carbon from [24]; cost constraint applied from [7]
manage	forest	natural forest management	used country-level biophysical potential as avoidable selective logging emissions in natural forests reported by Ellis *et al.* [10]; includes multiple forms of improved natural forest management: reduced-impact logging for climate (RIL-C), extended harvest cycles, increased post-harvest sequestration rates, and set-asides from logging activity; does not include avoidable illegal logging emissions; cost constraints derived from [7] and [25]
manage	forest (+savannahs)	avoided woodfuel	used country-level biophysical potential and cost constraint from [7]
manage	forest (+savannahs)	fire management	used country-level biophysical potential from [11]; applied cost constraint from [7]
manage	agriculture	trees in agricultural lands	generated new global and country-level estimates for the potential to incorporate trees into grazing lands (silvopastoral) and croplands (windbreaks and alley cropping) in forest and savannah biomes without reducing livestock or crop yields; baseline tree biomass in agricultural lands built from a recent pantropical 30 m biomass map [26]; potential additional growth rates from literature synthesis; adjusted cost constraints from [7]
manage	agriculture	nutrient management	generated new disaggregation to country level of global biophysical and cost-constrained estimate by Griscom *et al*. [7]
manage	agriculture	optimal grazing intensity	used country-level biophysical potential and applied cost constraint from [7]
restore	forest	reforestation	used data from [9] to extract country-level mitigation potential at US$100 Mg CO_2_^−1^ yr^−1^ threshold using spatially explicit pantropical marginal abatement cost curve model, calculated as mean annual additional sequestration over the time period 2030–2050; defined as shift from non-forest cover to forest cover at 30% tree-cover threshold; includes ‘afforestation’ with native trees
restore	wetland	peat restoration	used country-level biophysical potential and cost constraint from [7]
restore	wetland	mangrove restoration	generated new country-level estimates of biophysical potential; potential restorable mangrove area based on gross loss since 1996, subtracting area converted to urban land or lost to erosion [22], and is conservative (excludes potential restoration of mangroves lost before 1996); mean sequestration rate and cost constraints from [7]

‘Protect’ refers to pathways that prevent the loss of native ecosystems. ‘Restore’ refers to pathways that expand the spatial extent of native cover types, including forest and non-forest ecosystems, to areas from where they had previously been lost as a result of human activity. ‘Manage’ refers to pathways that avoid GHG emissions or enhance carbon sinks on working lands through improved management practices that do not reduce existing food, fibre or plant fuel yields (except where balanced by other pathways that increase yields). Note that, given our definition of the ‘restore’ category, we include, for example, the restoration of agricultural soil fertility and restoration of carbon stocks in degraded timber production forests within the ‘manage’ category. Pathways were constrained to be discrete (no double counting) and additional (change from a business-as-usual baseline). Safeguards were applied to avoid negative overall impacts on biodiversity and food and fibre security, as detailed by Griscom *et al*. [7]. We note that some implementation strategies can produce climate mitigation outcomes across more than one pathway and pathway type, such as forest certification, which can avoid both forest loss (via protection) and degradation (via improved management) [27].

### (b) Quantification of cost-effective mitigation

For all 12 pathways, we synthesized existing estimates, or derived new ones, for additional climate mitigation potential (i.e. potential change from business-as-usual behaviour). Our estimates represent average annual GHG removals and/or avoided emissions over two decades before mid-century (2030–2050) as countries move towards carbon neutrality. This allows for a ramp-up period between 2020 and 2030. We report ‘maximum with safeguards' biophysical mitigation potential (electronic supplementary material, tables S1 and S2) for all but one pathway: reforestation maximum mitigation potential, which is not available [9]. However, we focus our analysis on data we report for all 12 pathways: the portion of biophysical NCS potential for each pathway that could be delivered at an economically rational level, aligned with mitigation costs for other sectors, to limit warming to below 2°C (electronic supplementary material, table S3). We assumed, based on a literature review, that a maximum marginal cost of less than US$100 Mg CO_2_e (megagrams of carbon dioxide equivalent) in 2030 would be required across all sectors to hold warming below 2°C [28]. This is also considered the ‘social cost’ of carbon, or the mitigation cost threshold below which the cost of climate change to society is greater than the cost of mitigation [7]. With this threshold, we assigned low (30%), medium (60%) and high (90%) default cost-constrained mitigation levels, as a percentage of maximum-with-safeguards levels, informed by marginal abatement cost (MAC) curve, or related, literature detailed by Griscom *et al*. [7]. Our assignment of these default levels reflects that the MAC literature does not yet enable a precise understanding of the complex and geographically variable range of costs and benefits associated with NCS pathways. We used the results of a study employing more refined methods for the reforestation pathway [9], a pantropical spatially explicit MAC curve, from which we extracted national reforestation mitigation potential at the US$100 cost threshold. We were unable to use the equivalent cost-constrained information on ‘deforestation’ available from this source for our ‘avoided forest conversion’ pathway. We constrained ‘avoided forest conversion’ to exclude forest loss that does not represent land use conversion activity (e.g. forest cover clearing cycles of swidden-fallow cultivation). Such activities were included as ‘deforestation’ reported by Busch *et al.* [9]. We refer to the sum of these 12 pathways, with food security and biodiversity safeguards described below, and constrained below the social cost of carbon (less than US$100 per Mg CO_2_e), as ‘cost-effective NCS.’

### (c) Data sources

For seven of the 12 pathways, we used country-level biophysical potential from prior studies, with adjustments we made to harmonize accounting, in particular, to avoid double counting. For two pathways, we generated new country-level disaggregations of prior global estimates (avoided forest conversion, nutrient management). For three pathways, we generated new global and country-level estimates (trees in agricultural lands, avoided mangrove loss, mangrove restoration). See table 1 for more detailed summary of methods and sources used for each pathway. See additional methods details in the electronic supplementary material.

### (d) Geographical area

We analysed all tropical countries, defined as those having greater than 50% of their land area between 23.5°N and 23.5°S. We excluded countries with less than 10 000 km^2^ of land area owing to insufficient resolution in our datasets, with the exception of our new mangrove data which we report for smaller countries (see electronic supplementary material, table S2).

### (e) National factors affecting the feasibility of implementing NCS

We considered three factors that could affect the pace and extent of NCS implementation by national governments: (i) the proportional contribution of NCS to balancing a nation's GHG emissions and removals, (ii) political governance, and (iii) the economic cost of, and potential revenues from, NCS relative to current economic activities. We compared national NCS potential with ‘current’ total national GHG emissions, which we define as mean annual GHG emissions during the most recent 5 year period for which historic data is available (2010–2014) on total national anthropogenic GHG emissions, including the AFOLU sector, as reported by CAIT (http://cait.wri.org) and derived from the United Nations Framework Convention on Climate Change (UNFCCC) Secretariat. We also compared NCS potential with emissions reduction targets reported in NDCs (for those countries reporting fixed targets). To address the governance factor, we calculated the mean of six Worldwide Governance Indicators (www.govindicators.org) for each country: voice and accountability, government effectiveness, political stability and non-violence, regulatory quality, rule of law, and control of corruption. We considered these as likely to indicate the extent to which governance may enable or limit the development and/or implementation of NCS policies [17]. Links have been shown between governance and effective deployment of climate change mitigation activities [29].

To assess the ability of a country to finance its NCS potential, we compared national NCS potential, valued at US$50 per Mg CO_2_e, with gross domestic product (GDP). Given the MAC threshold of US$100, we assumed a US$50 per Mg CO_2_e value for NCS would best approximate the mean cost of delivering NCS.

## 3. Results and discussion

### (a) Pantropical climate mitigation potential of NCS

The 12 NCS pathways we consider here could deliver 6.56 Pg CO_2_e yr^−1^ across 79 tropical countries and territories between 2030 and 2050 at ‘cost-effective’ levels (less than US$100 per Mg CO_2_e). All of these NCS pathways also deliver other important benefits, including biodiversity, flood control, water filtration, air filtration and soil fertility [7]. Summing across the 76 countries that have national GHG emissions reporting data available, we estimate that cost-effective NCS (6.51 Pg CO_2_e yr^−1^) has the potential to mitigate nearly half (48%) of recent national historic annual GHG emissions across all sectors (13.68 Pg CO_2_e yr^−1^). In over half of these tropical countries (*N* = 39), cost-effective NCS could mitigate over half of recent historic national emissions. In more than a quarter of these countries (*N* = 22), cost-effective NCS potential is larger than recent historic national GHG emissions. Since NCS both avoid existing AFOLU emissions and enhance terrestrial carbon sinks, these countries could become net carbon sinks. For all 58 tropical countries with quantified NDC targets as of 2019, the aggregate cost-effective NCS potential (5.72 Pg CO_2_e yr^−1^) is larger than the aggregate emissions reductions implied by their current NDC targets, conditional and unconditional, for all sectors (4.2 Pg CO_2_e yr^−1^). Likewise, cost-effective NCS could deliver more than the total national NDC target for most (79% of) tropical countries with quantified NDC targets.

If cost-effective NCS are fully implemented by 2030, this mitigation potential will not begin to decline (or saturate) before mid-century; however, NCS can be expected to mitigate a declining proportion of total national GHG emissions if national GHG emissions from other sectors continue to increase. On the other hand, our estimate does not include eight of the 20 NCS pathways, which contribute 14% of total global NCS potential [7]. Also, our estimates for the largest NCS pathways are conservative compared with other recent assessments. Our aggregate cost-effective reforestation estimate (1.2 Pg CO_2_e yr^−1^) derived from [9] is 48% below the pantropical portion of the global cost-effective estimate reported by Griscom *et al*. [7], which itself is only one-third as large as a more recent estimate [30]. Our cost-effective pantropical estimate for avoided forest conversion is more conservatively defined, and lower, than a recent cost-constrained estimate for avoided deforestation [9]. We are aware of no comparable estimates for the third largest pathway: adding trees to agricultural lands. Our estimate of the fourth largest pathway, avoided peat impacts derived from [7], does not yet include the avoided loss of vast peat forests recently reported in the Congo Basin [31]. Further, our estimates do not fully account for the potential economic benefits of ecosystem services mentioned above, which can be very high [32]. While our estimates improve on prior pantropical estimates of NCS potential [7], national (e.g. [33]) and sub-national (e.g. [34,35]) NCS assessments are needed, allowing better consideration of national and local circumstances, and in some cases the availability of better national and regional datasets.

### (b) Variable contribution of pathway types to national NCS portfolios

**Figure 1.**
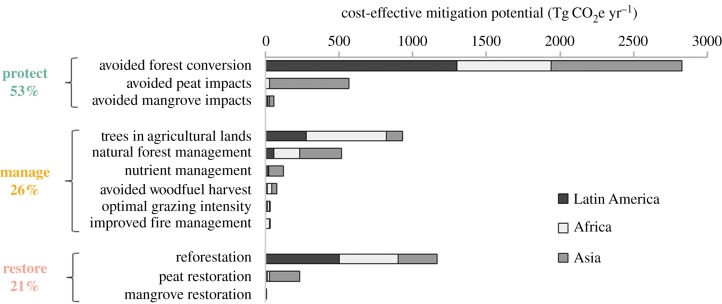
Pantropical climate mitigation potential of three types of NCS pathways (protect, manage, restore), and 12 individual pathways, across three tropical regions (Latin America, Africa, Asia), constrained to ‘cost-effective’ levels (less than US$100 per Mg CO_2_e). The percentage of total mitigation potential is reported on the left for each type of pathway. (Online version in colour.)

NCS potential in tropical countries involves a broad mix of protection, improved management and restoration pathways. More than half of cost-effective NCS, totalling 3.5 Pg CO_2_e yr^−1^ (figure 1), involve some form of protection through the avoided conversion of non-wetland forests (43%) and avoided impacts to peatlands and mangroves (10%). We estimate that the largest individual pathway, avoided forest conversion, offers more than twice as much of the cost-effective climate mitigation potential as the second largest pathway (reforestation, figure 1). This reflects high recent tropical forest loss rates [13], and relatively low economic costs of avoiding such losses [9]. Protection pathways also consistently offer the most diverse set of biodiversity and ecosystem service benefits [7]. Activities that can ‘protect’ lands, which is to say reduce rates of intact ecosystem conversion to other land uses, include establishing any one of the IUCN protected area categories [36,37], improving indigenous and community land tenure [38], and voluntary incentives [39].

One-fifth of cost-effective NCS involve the restoration of native forest and wetland cover (totalling 1.4 Pg CO_2_e yr^−1^, figure 1). These ‘restore’ pathways invoke the largest trade-offs with business-as-usual land uses, since relatively large areas would need to be taken out of current use and returned to native cover per tonne of climate mitigation outcome [40]. For example, tropical reforestation may typically sequester about 3 Mg C ha^−1^ yr^−1^ [9] while avoiding tropical forest conversion will typically avoid emissions of about 100 Mg C ha^−1^ yr^−1^ [7]; hence in a given year considerably more land area is needed for reforestation to generate the same climate mitigation outcome as avoided forest conversion. While we assume most restoration opportunity lands are currently in use, there are extensive tropical lands where opportunity costs of restoration are low [9]. Restoration geographies targeting low opportunity cost [9] and high conservation value [40], and aligned with support for smallholders and sustainable intensification, can achieve favourable conditions for long-term restoration of native ecosystems [41]. While reforestation (including afforestation in forest ecoregions) would displace some lower intensity agricultural production systems in particular grazing lands, this can be achieved while feeding a growing human population with shifts towards healthier plant-based diets that reduce healthcare costs [42], technological advances that promote agricultural intensification, and/or the advance of cultured meat technology [43]. Reforestation can also generate additional global commodities and economically valuable services (e.g. fibre, fuel, drinking water, flood control, fisheries) [12]. Restoration of diverse native ecosystems is particularly important for wetland systems, which are much less extensive in area than reforestation opportunities yet deliver the highest carbon storage and other ecosystem services per hectare restored [32].

One-quarter of total cost-effective NCS are provided by pathways employing improved management practices, totalling 1.7 Pg CO_2_e yr^−1^. These ‘manage’ pathways involve lower climate benefits per hectare and require the most extensive area of engagement [4], relative to both ‘protect’ and ‘restore’ pathways. On the other hand, ‘manage’ pathways do not require a change in land use or yields. Rather, ‘manage’ pathways can improve land production systems to increase the sustainability of food, fibre and fuel production while delivering a variety of other ecosystem services [7]. ‘Manage’ pathways can avoid challenges associated with land use change often confronting ‘protect’ and ‘restore’ pathways, such as the potential need for alternative livelihoods when reforesting grazing lands or halting the conversion of forest to agriculture. The largest of the ‘manage’ pathways in the tropics is ‘trees in agricultural lands,’ and includes various agroforestry and silvopastoral systems. While these and other improved management activities are constrained to those that maintain or increase agricultural or forestry product yields, some assume improved technologies and/or outcomes of other pathways to meet demands. Specifically, the second largest ‘manage’ pathway, natural forest management, includes reduced-impact logging for climate (RIL-C) which reduces logging emissions while maintaining or increasing long-term timber yields [10]; however, it also includes extended harvest cycles, which assume that increased wood yields from the reforestation pathway make up for deferred yields from existing native production forests. The nutrient management pathway reduces N_2_O emissions by avoiding the over-application of nitrogen fertilizer, through the use of best practices and/or improved technologies [7].

**Figure 2.**
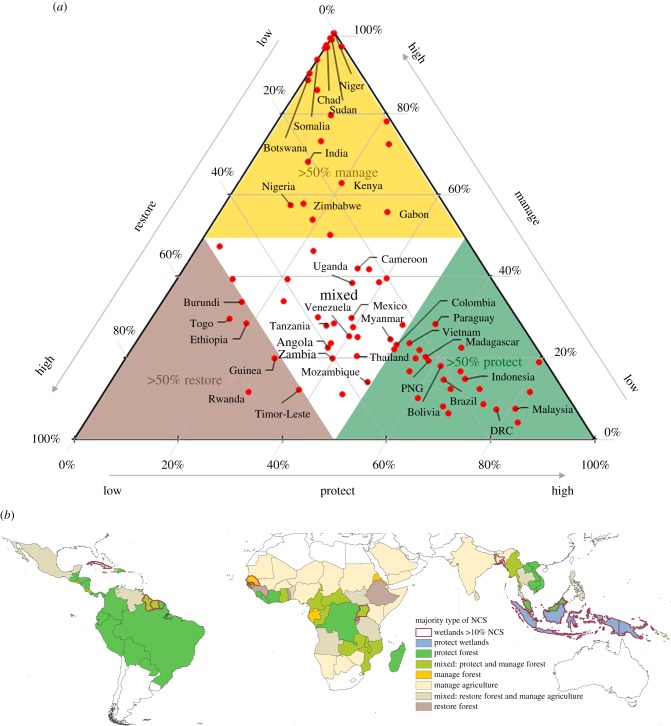
Countries are classified into one of three groups (‘protect,’ ‘manage’ or ‘restore’) depending on the pathway type contributing the majority (greater than 50%) of mitigation potential—or into a fourth group (‘mixed’) if no pathway type contributed the majority (*a*). For example, Zambia is ‘mixed’ (40% of total NCS are ‘protect’ pathways, 40% ‘restore’ and 20% ‘manage’). The 10 countries with the largest total NCS potential in each group are identified by name in (*a*). NCS countries are further classified into seven groups based on the cover type (forest, agriculture, wetland) of pathways contributing the most mitigation potential (*b*). Red outlines identify countries where greater than 10% of NCS is from wetland pathways that deliver multiple ecosystem services. See table 1 for individual pathways associated with each country group. DRC, Democratic Republic of the Congo; PNG, Papua New Guinea.

The number of countries in which the majority (greater than 50%) of NCS potential falls into one of the three pathway types (protect = 25 countries, manage = 23, restore = 6) follows the rank order of aggregate mitigation potential (above); however, a large number of countries (*N* = 25) have a more balanced NCS portfolio (i.e. ‘mixed,’ figure 2*a*).

Protection pathways offer the majority of NCS opportunity in countries across much of Central and South America and Southeast Asia. Countries in Africa and the northern portion of tropical Asia have more variable NCS portfolios (figure 2*b*). Protection and forest management are majority opportunities in Congo Basin countries, while restoration and improved management of agricultural lands are majority opportunities to the north and south of the Congo Basin. Despite these regionally distinct NCS portfolios, avoided forest conversion is the largest individual pathway in all three tropical regions (figure 1). All but one of the countries where restoration (primarily reforestation) is the majority NCS opportunity are in Africa (‘restore’ countries in figure 2*a*); however, the largest absolute reforestation mitigation potential is in Latin America (figure 1).

Wetland pathways do not hold the majority of NCS in any of the 79 tropical countries or territories, although avoided peat impacts are the largest individual pathway in Indonesia and Papua New Guinea. Indonesia alone holds 76% of pantropical wetland mitigation potential (combining peat and mangrove pathways). At national scales, wetlands usually cover much less area than other ecosystems, yet they play a disproportionate role in the provision of ecosystem services and associated SDGs [12]. We highlight 12 countries, across all tropical regions, where wetland protection and restoration offer a relatively large contribution to national NCS portfolios (greater than or equal to 10%) (figure 2*b*). We also report data on opportunities to avoid the loss of and to restore mangroves in small island nations and territories, where mangroves can play a disproportionate role in national climate mitigation (electronic supplementary material, table S2).

### (c) Alignment with national monitoring and verification

The updates to pantropical NCS estimates that we synthesize here reflect the rapid advance of scientific measures to improve estimates for pathways that have had greater uncertainty (i.e. reforestation, natural forest management, fire management, trees in agricultural lands and mangroves [7]). There is an additional challenge of aligning improved pantropical data with national measurement, monitoring and verification systems. Even for pathways with relatively well-constrained uncertainty and freely available spatially explicit annual monitoring systems, such as avoided forest conversion, there can be poor alignment between global datasets and national reporting [13]. On the other hand, some new pathway estimates reported here are generally not included in national monitoring systems, avoid double counting with pathways that are included in national monitoring systems, and could simply be added to national monitoring and verification systems using existing robust carbon verification standards (e.g. improved natural forest management [10]).

### (d) National factors affecting the feasibility of implementing NCS

**Figure 3.**
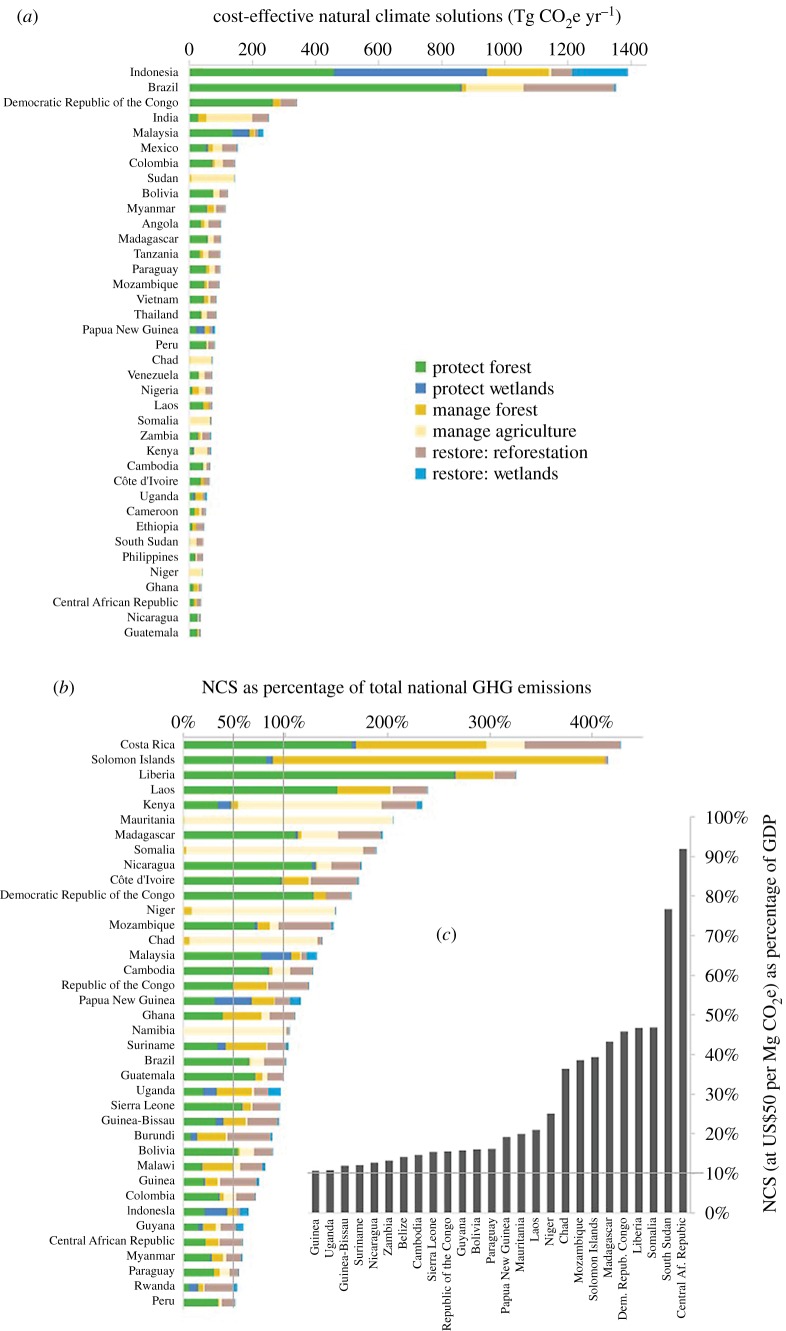
(*a*) Top 40 tropical countries in terms of total cost-effective NCS (sum of 12 NCS pathways). (*b*) Tropical countries in which cost-effective NCS potential is equivalent to 50% or more of current total national GHG emissions, and (*c*) tropical countries where NCS (assuming implementation cost at US$50 per Mg CO_2_e) represent greater than 10% of gross domestic product (GDP). (Online version in colour.)

While a small subset of relatively large countries harbours the majority of pantropical NCS potential, opportunities to unlock NCS in the near term are widely distributed across the tropics. Among the 79 tropical countries and territories, four large countries (Indonesia, Brazil, Democratic Republic of the Congo and India) hold over half (53%) of pantropical cost-effective NCS potential, and 80% of the potential is held by the top 20 countries—ranked by NCS potential (figure 3*a*).

Factors affecting the feasibility of implementing NCS vary widely. Among the top 20 countries in figure 3*a* (Indonesia to Chad), the percentage of recent historic national GHG emissions that can be mitigated by cost-effective NCS, fundamental to determining the role of NCS in NDCs, ranges from 9% in India to 195% in Madagascar. Only four of these top 20 countries (in figure 3*a*), in terms of the overall potential of cost-effective NCS, are also among the 20 countries with the highest NCS potential in proportion to historic national GHG emissions (figure 3*b*). Hence, the absolute size of national NCS is not a good indicator of the proportional contribution of NCS to balancing a country's GHG emissions with removals.

Though the social cost of carbon is considered US$100 per Mg CO_2_e, the financial capacity of countries to implement up to this level of NCS potential is highly variable, and not financially feasible for many (figure 3*c*). For example, among the four countries with the largest NCS potential, the costs of implementing cost-effective NCS (assuming an average cost of US$50 per Mg CO_2_e, see material and methods), ranges from 46% of GDP in the Democratic Republic of the Congo to less than 1% in India. The median cost of implementing cost-effective NCS is equivalent to 5.5% of national GDP for tropical countries. Cost-effective levels of NCS implementation are equivalent to less than 1% of GDP for 15 tropical countries, and to over 10% of GDP for 26 tropical countries (figure 3*c*). Implementation of cost-effective NCS could mitigate over half of the national GHG emissions for most (88%) of these 26 countries with the most limited capacity to finance NCS (figure 3*b*,*c*).

**Figure 4.**
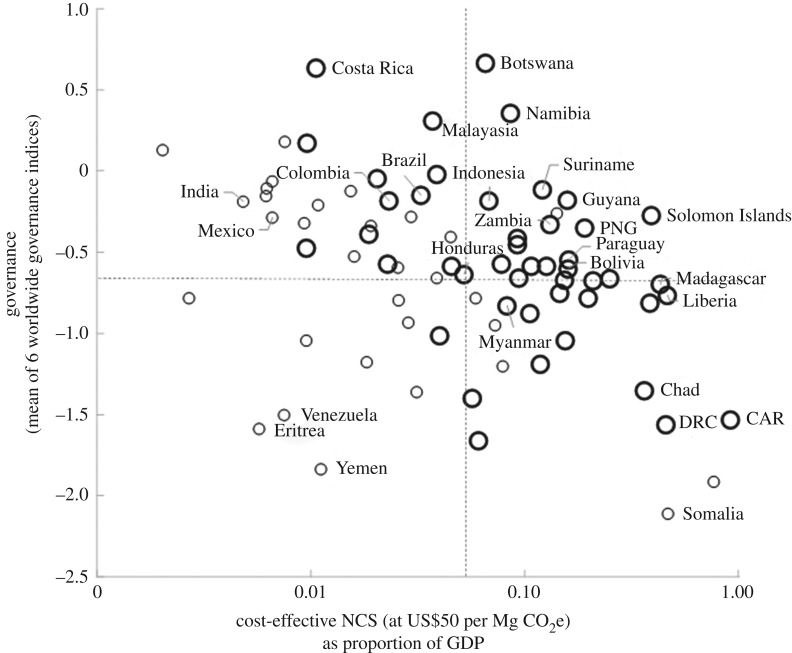
Tropical countries (circles) are displayed with respect to governance (*y*-axis), as reflected by the mean of the six Worldwide Governance Indicators, and cost-effective NCS (sum of 12 pathways, assuming mean implementation cost of US$50 per Mg CO_2_e) as percentage of GDP (*x*-axis, displayed as logarithmic). Larger circles identify countries where cost-effective NCS is greater than 50% of total national GHG emissions (versus countries where NCS is less than 50% of GHG emissions identified by smaller circles). Countries in the upper-right section of this graph, with above average (−0.61) governance and where NCS > 5% of GDP, may indicate opportunities for international investment in NCS, particularly those with larger circles. CAR, Central African Republic; DRC, Democratic Republic of the Congo; PNG, Papua New Guinea.

We also considered the Worldwide Governance Indicators as metrics for the feasibility and capacity of countries to successfully implement NCS. As the GDP impact of implementing cost-effective NCS increases, governance indicators tend to decline (figure 4; *R*^2^ = 0.12, *F*_1,76_ = 10.49, *p* = 0.002). While this correlation is significant, it is weak. A number of countries with above-average governance (greater than −0.61) also have relatively limited financial capacity to implement NCS opportunities (NCS at US$50 greater than 5% of GDP), and nearly all of these countries have large NCS opportunities relative to national GHG emissions (greater than 50%), indicating that financing mechanisms could be particularly effective in these geographies (upper-right section of figure 4).

## 4. Conclusion

Our results clarify the central role of NCS for most tropical countries to deliver on both existing national commitments and future higher ambition NDCs and to balance emissions with removals by mid-century. Our analysis identifies countries that are of particular interest for unlocking this large tropical NCS potential. Most obviously, there are a small set of large to medium-sized countries that harbour the majority of tropical NCS opportunity and that have high governance indicators and strong to intermediate financial capacity, relatively speaking (i.e. Indonesia, Brazil, India, Malaysia, Mexico, Colombia, figures 3*a* and 4). International co-financing could accelerate NCS implementation in such countries, which is critical given the sheer magnitude of their NCS potential. Provided that access to finance is matched by political will (where indicated by ambitious NDCs) and institutional capabilities (where indicated by strong and improving governance) there is reason for optimism that these national governments are capable of unlocking a great deal of their cost-effective NCS potential. Despite the large NCS potential in these countries, it is also critical that they take steps to de-carbonize their significant industry and energy infrastructure in order to balance emissions with removals.

Another group of countries, including for example Botswana, Namibia, Guyana, Suriname and the Solomon Islands, has above-average governance indicators but considerably less financial capacity to implement NCS than the countries mentioned above (see the upper-right section of figure 4). Nearly all of these more financially constrained countries have cost-effective NCS potential that can mitigate over half of their national GHG emissions. Increased international investment to help unlock NCS among this group could advance progress towards SDGs domestically while balancing emissions with removals domestically, and also delivering removals for other countries that do not have a large carbon sink potential. If international financial support is used for socially positive approaches to NCS implementation, it could have large positive impacts on these economies while delivering SDGs and significant global climate mitigation.

A number of other countries with below-average governance indicators share similar financial constraints and a disproportionate role of NCS for balancing emissions with removals (e.g. Democratic Republic of the Congo, Central African Republic and Myanmar, in the lower-right section of figure 4). These countries may require longer-term support to address governance challenges while investing in NCS opportunities.

Most countries with below-average governance indicators and relatively lower financial constraints on implementing available NCS opportunities are also countries where NCS offers a minority of the solution set for balancing emissions with removals (e.g. Eritrea, Yemen, Saudi Arabia, and other smaller circles in the lower-left section of figure 4). These countries present much more limited opportunities for international financial support of NCS to accomplish multiple outcomes described above. However, there are some countries in this group, like Venezuela, that do hold significant NCS potential (figure 3*a*) that should not be ignored.

The Worldwide Governance Indicators identify broad trends among countries; however, these indices are coarse measures and can mask critical differences among countries. One limitation is the indicators' emphasis on country capacity, with less emphasis on commitment [44]. This analysis is intended to set the stage for more in-depth consideration of complex national and sub-national circumstances as needed to make careful judgements, both internationally and domestically, about prioritizing financial and governmental support for implementing NCS while achieving associated SDGs.

In addition to these broad governance, biophysical and financial factors that influence the feasibility of implementing cost-effective NCS potential, we also looked at how NCS can be implemented in each country by considering three types of NCS pathways (‘protect,’ ‘manage’ and ‘restore’) that present distinct opportunities and challenges for engaging both local and national stakeholders as discussed above. While each tropical country and region has a distinct NCS portfolio, in terms of the relative magnitude of climate mitigation available from protection, management and restoration of different biomes, most countries have opportunities across all three types of NCS pathways. Decisions about which options to include or enhance in updating NDCs will depend on national circumstances and preferences. To inform those decisions, we provide here a classification of tropical NCS country groups, identifying the countries where specific types of NCS opportunities provide the majority of mitigation potential (figure 2). This country classification, and the groups of countries we identify based on governance and financial constraints (figure 4), could be used to facilitate learning networks across countries with a similar set of NCS opportunities and challenges.

This approach to identifying majority NCS opportunities will have greater local relevance if extended to sub-national regions, which can have very different NCS opportunities, particularly across large countries. For example, while the majority NCS pathway type in Brazil is ‘protect,’ this national classification masks the globally significant reforestation opportunities that exist in southern regions of Brazil [9]. Further research is also needed to improve our understanding of region-specific costs and benefits of implementing different types of NCS, and to align NCS priorities with the full range of SDGs [12] and other country-specific feasibility considerations.

More broadly, our intent is to provide government officials, the private sector and civil society with information to advance an ambitious vision for unlocking the large potential of NCS in the tropics. Indeed, such a vision is required if we are to make transformational change across the tropics to achieve current and future NDC targets while delivering SDGs. Globally, both decarbonization of energy and industry and NCS are necessary for achieving Paris Agreement goals [3,4]. For many countries in the tropics, the emphasis should be on NCS for delivering enhanced NDCs and globally significant contributions to the Paris Agreement needed to avoid catastrophic climate change.

## Supplementary Material

Methods Details

## Supplementary Material

Supplementary tables

## Endnote

1 The text of the Paris Agreement is available at https://unfccc.int/sites/default/files/english_paris_agreement.pdf.

## Data accessibility

This article has no additional data.
