# Supplementary material for: National mitigation potential from natural climate solutions in the tropics
Source: Philos Trans R Soc Lond B Biol Sci. 2020 Jan 27;375(1794):20190126. doi: 10.1098/rstb.2019.0126 (PMC7017762; doi:10.1098/rstb.2019.0126)
Supplement: Methods Details [file rstb20190126supp1.docx]

**Supplementary material: Methods Details**

for

**Title:** National mitigation potential from natural climate solutions in the tropics

**Authors:** Bronson W. Griscom, Jonah Busch, Susan C. Cook-Patton, Peter W. Ellis, Jason Funk, Sara M. Leavitt, Guy Lomax, Will Turner, Melissa Chapman, Jens Engelmann, Noel P. Gurwick, Emily Landis, Deborah Lawrence, Yadvinder Malhi, Lisa Schindler Murray, Diego Navarrete, Stephanie Roe, Sabrina Scull, Pete Smith, Charlotte Streck, Wayne S. Walker, Thomas Worthington

*Reforestation*

We extracted total reforestation mitigation potential per country at the marginal abatement cost of 100 USD per MgCO_2_ from spatially explicit pantropical marginal abatement cost curves [1]. This new pantropical dataset does not explicitly include the food security safeguards and deductions for double-counting with other pathways described here. However, mitigation levels extracted from [1] at the 100 USD per MgCO_2_ threshold are lower than those reported at the same cost threshold by [2] who included a spatially explicit deduction for food security and conservative deductions to avoid double-counting with other pathways (e.g. grazing-optimal intensity). Hence, we conclude that additional deductions for double-counting and food security safeguards – as defined by [2] and used here – are not necessary to apply to the considerably more refined econometric analysis by Busch et al. [1].

*Avoided Forest Conversion*

We updated regional estimates of avoided forest conversion emissions [2] by improving estimates of soil carbon fluxes, and correcting source data errors [3]. We disaggregated our estimates to individual countries by proportionally allocating our regional estimates to countries in each region based on country-level forest carbon loss estimates from Global Forest Watch Climate (<http://climate.globalforestwatch.org/>, [4]). This approach allowed us to retain the exclusion of forest loss fluxes from “managed forests” as reported by [5], which avoids double-counting with other pathways, in particular, Improved Natural Forest Management.

*Improved Natural Forest Management*

We updated the Griscom et al. [2] estimate using Ellis et al. [6], who estimate country-level baseline pantropical selective logging emissions and the portion of these emissions that could be avoided through implementing reduced-impact logging for climate practices (RIL-C). Other forms of improved natural forest management can avoid or offset the remaining portion of selective logging emissions through mid-century, including extended harvest rotations, increased post-harvest sequestration rates (e.g. removal of super-abundant competing non-tree vegetation like lianas), and designation of set-aside areas for protection from logging activity [7]. Where timber yields are delayed (ie. extended rotations) or reduced (ie. set-asides), we assume that timber volumes are made-up by a limited portion of additional wood yielded through the reforestation pathway. We assumed 90% of mitigation potential can be delivered at a marginal abatement cost of 100 USD per MgCO_2_^-1^ for avoided emissions from RIL-C based on [8], and for all other forms of improved forest management we followed [2] to assume 60% can be delivered at the 100 USD threshold.

*Trees in Agricultural Lands*

We calculated mitigation potential of trees in croplands in each country by combining the methodological approach in [2] with a new data layer of tree biomass in agricultural lands. In developing this new spatial dataset, we expanded this pathway to include the addition of trees in grazing lands, in order to explore the magnitude of opportunity for silvopastoral systems to maintain grazing systems while capturing additional carbon. We considered three different categories of practices for including trees in agricultural lands (croplands and grazing lands): windbreaks, alley cropping, and silvopastoral systems. Windbreaks involve a line of trees along field margins which can help reduce soil erosion, evapotranspiration, and wind stress to crops, improving yields in some, but not all, cases [9–12]. Alley cropping refers to planting annual crops between trees, and silvopastoral systems incorporate trees into grazing lands. We assumed that expansion of these activities would be suitable in a portion of current crop and grazing lands that have low existing tree biomass.

Croplands with low woody biomass are potential targets for the addition of trees to croplands. To find them, we began with a global extension of the 30-m biomass map produced by [13] combined with the latest 30-m global cropland layer (GFSAD). Following Zomer et al. [14] we defined systems with low woody biomass as those with less than 5 MgC ha^-1^. To find grazing lands with low woody biomass, we combined the same 30-m biomass map with a 1-km global “pasture” layer [15]. Whenever cropland and grazing land pixels overlapped, we gave priority to the higher resolution and more recent cropland layer and counted the associated biomass as residing within cropland. We then used a 30-m resolution global forest cover dataset to filter out areas with > 25% tree cover [16]. We also removed rural and urban settlements by excluding areas with > 750 people per km2 in 2015 using the Gridded Population of the World (GPW, v4). The product was a global map of baseline tree biomass carbon density in croplands and grazing lands.

We then estimated the climate mitigation benefit of adding trees to croplands and grazing lands in forest and savanna biomes with low woody biomass (< 5 MgC ha^-1^), without disrupting existing yields. We assumed that existing yields would not be diminished across 22% of croplands for the addition of trees in the form of alley cropping [17], and across 50% of croplands for the addition of trees in the forms of windbreaks [2], for all cropland that occurred in forest or savanna biomes [18]. Likewise, we assumed that silvopastoral systems would not reduce livestock yields across 50% of low woody biomass grazing lands in both forest and savanna biomes. We assumed zero potential for additional tree cover in native grassland biomes.

We were not concerned about double-counting between this pathway (trees in agricultural lands) and the reforestation pathway, given that the cost-constrained reforestation we report here (from [1]) targets returning lands to forests where agricultural yields are either marginal or not present (i.e. land with the lowest opportunity cost). Likewise, the cost-constrained reforestation potential we report here summed for tropical countries (1.2 PgCO_2_e yr^-1^) is a fifth of the “maximum with safeguards” reforestation potential summed across the same countries (5.6 PgCO_2_e yr^-1^) reported by Griscom et al. [2], despite their additional exclusion of reforestation in croplands. In contrast, the addition of trees to agricultural lands we report here targets the subset (50% or less, prior to cost constraints) of productive agricultural systems where an investment in adding trees is desired to improve sustainable yields and/or increase carbon storage.

We conducted a literature review to identify average carbon sequestration rates for each of these agroforestry practices. We assumed sequestration in combined biomass and soil carbon of 1.2 MgC ha^-1^ yr^-1^ for alley cropping and 0.175 MgC ha^-1^ yr^-1^ for windbreaks based on references synthesized by [2]. For silvopastoral systems, we reviewed the literature from Costa Rica, Ecuador, Ethiopia, India, Mexico and Nicaragua, and estimate that the median sequestration rate across silvopastoral systems is 1.88 MgC ha^-1^ yr^-1^. This rate includes above and belowground biomass. Where belowground biomass was not included in individual sources cited above, we added it using the average root:shoot ratio in woodland systems [19]. Because conversion from grazing lands to more forested ecosystems can have a range of impacts on soil organic carbon from net gain to net loss (unlike the carbon sequestration rates for alley cropping and windbreaks; [20]), we conservatively assumed no net carbon accumulation in the soil for silvopastoral systems. We also note that we did not include additional mitigation potential from farmer managed natural regeneration (FMNR), an approach to adding trees in agricultural lands in dryer areas within a number of countries in Africa. We made this conservative omission due to the lack of data on dryland crop area in African countries, to reflect our concerns about methods consistency across tropical regions, and to avoid double-counting.

To estimate annual mitigation potential for each agroforestry practice in each country we multiplied the area of opportunity by the appropriate carbon sequestration rate as described above.

*Nutrient Management*

We disaggregated the global estimate (5) of the mitigation potential for improved cropland nutrient management practices as follows. We estimate the opportunity to reduce nitrous oxide emissions from global cropland soils from optimization of the application of synthetic nitrogen fertilizers without adversely impacting crop yield. We first estimate business-as-usual nitrogen fertilizer consumption in 2030 based on projections for twelve world regions by Lassaletta et al. [21], assuming that the share of total fertilizer use in each country in a region remains constant at average levels in the period 2002-2009 (FAO 2018). We then compile estimates of average nitrogen use efficiency (NUE) in each country - the ratio of total nitrogen inputs to nitrogen harvested in products - for the same period based on country-level data by Zhang et al. [22] and Lassaletta et al. [23].

To model the mitigation opportunity, we estimate the savings in synthetic nitrogen fertilizer use achievable by raising NUE from business-as-usual values (assumed to be equal to current values) to regional target values proposed by [22], ranging from 60% in China to 75% in Europe and North America. We assign zero potential where current NUE exceeds the regional target value, since this likely reflects either existing efficient practices or mining of soil nitrogen [22]. Finally, we translate the reduction in fertilizer consumption to a reduction in N_2_O emissions reduction assuming a total emissions factor of 0.0254 kg N emitted as N_2_O per kg N applied [24], and convert this into CO_2_-equivalent terms using the 100-year nitrous oxide global warming potential [25].

*Avoided Mangrove Loss*

Griscom et al. [2] include both seagrass and mangroves in estimating an “avoided coastal wetland impacts” pathway. Here, as reflected by the pathway name, we only include the larger mangrove component due to the lack of pantropical data on seagrass that is sufficiently robust and spatially explicit for national estimates. We extracted mangrove extents from the Global Mangrove Watch (GMW) (<http://data.unep-wcmc.org/datasets/45>; [26]) for 1996, 2007, 2010, and 2016 timesteps to calculate rates of mangrove loss. The mangrove footprint was split into 6,274 individual typological units based on their proximity to coastal geomorphological features [27]. Transboundary typological units were assigned to the country within which the majority of the unit’s area was found (with the exception of the Sundarbans, which was split between India and Bangladesh based on the international border).

To calculate the mangrove biomass and soil carbon values, we began by deriving the maximum mangrove footprint as the union of all timestep extents from the Global Mangrove Watch. We used country level data on the mean aboveground biomass (AGB) (Mg ha^-1^) of mangroves from Simard et al. ([28]; Supplementary Table 2 - 6). This was converted to mean AGB carbon using the stoichiometric factor of 0.451 [28], and then to mean AGB CO_2_ using a conversion factor of 3.67 [29]. It should be noted that there was a mismatch in the spatial extent of the mangrove footprint between the Simard et al. [28] dataset and the GMW dataset used to examine loss; however, [28] represents the most up-to-date and consistent assessment of mangrove AGB.

We used a global map of soil organic carbon at 30 m resolution [30] for mangrove soil organic carbon (SOC). The data were aggregated to national level statistics. As with the AGB, the SOC data was based on the mangrove footprint of Giri et al. [31] rather than the updated GMW mangrove extents. SOC was converted to SOC CO_2_ using a conversion factor of 3.67 [29]. Based on the baseline maps of SOC, changes in soil carbon over time were estimated based on changes detected in remote sensing data assuming a quasi-steady state over the period.

Mangrove loss over the study period was assessed by subtracting the 2016 extent from the maximum mangrove footprint (the union of the 1996, 2007, 2010 and 2016 timesteps) and therefore represents gross loss without accounting for gains or regeneration (i.e. net loss). This gross loss was converted to an annual rate for the 20-year period. Yearly avoided CO_2_ loss was the sum of SOC and AGB CO_2_ multiplied by the annual gross loss rate.

*Mangrove Restoration*

As with the Mangrove Loss pathway above, we re-named this pathway from “Coastal Wetland Restoration” described in [2] to reflect the exclusion of seagrass. The potential restorable mangrove area in each typological unit was calculated by subtracting the area converted to urban land, or eroded, from the area of gross loss (as described above). As such, this is a conservative estimate of mangrove restoration potential because it does not include areas where mangroves were lost prior to 1996. Restorable area at the scale of individual typological units was aggregated to country level statistics.

Urban areas, defined as human-made building structures with a vertical component, were identified from the Global Urban Footprint (GUF) dataset [32], and areas of gross loss that intersected with the GUF were classed as urbanized. Areas of erosion were identified using a combination of the extent of global mudflats for the year 2016 [33], extent of bare ground [16], and water occurrence change intensity [34]. The bare ground data were derived from Landsat 7 ETM+ cloud-free composites to estimate the minimum percentage of bare ground per pixel for the circa 2010 peak growing season [16]. For this analysis, a pixel was classified as bare if it had ≥50% bare ground. Water occurrence change was calculated by matching monthly observations between two time periods (1984-1999 and 2000-2015) and identifying percentage change in water presence [34]. We identified areas within a 100m buffer of the coastline (coastline from a modified version of GADM) that had had a ≥20% increase in water intensity between the two time periods. Areas of erosion were identified by overlaying mudflat presence, extent of bare grounds, and water occurrence change on top of the areas of mangrove gross loss. Erosion was assumed in those loss areas where water occurrence changed intensity or mudflats were present. In addition, areas were assigned to erosion if loss areas were overlaid by combinations of two or more of the following layers: mudflats, bare ground, and/or water occurrence change in intensity. Areas of erosion were removed if they intersected with the GUF or layers representing Global Tree Canopy Cover for circa 2010 [16] or Global 30m Cropland Extent (<https://www.croplands.org/app/map?lat=0.17578&lng=0&zoom=2>).

Potential benefits from restoration were calculated by multiplying the country-level area of restorable mangroves by a global carbon sequestration value of 6.4 [2] and converting to CO_2_ equivalent using a conversion factor of 3.67 [29].

**References**

1. Busch J, Engelmann J, Cook-Patton SC, Griscom BW, Kroeger T, Possingham H, Shyamsundar P. 2019 Potential for low-cost carbon dioxide removal through tropical reforestation. *Nat. Clim. Chang. 2019 96* **9**, 463. (doi:10.1038/s41558-019-0485-x)

2. Griscom BW *et al.* 2017 Natural climate solutions. *Proc. Natl. Acad. Sci.* **114**, 11645–11650. (doi:10.1073/pnas.1710465114)

3. Tyukavina A, Baccini A, Hansen MC, Potapov P V, Stehman S V, Houghton RA, Turubanova S, Goetz SJ. 2018 Corrigendum: Aboveground carbon loss in natural and managed tropical forests from 2000 to 2012. *Environ. Res. Lett.* **13**, 109501. (doi:10.1088/1748-9326/aae31e)

4. Zarin DJ *et al.* 2016 Can carbon emissions from tropical deforestation drop by 50% in five years? *Glob. Chang. Biol.* **22**, 1336–47. (doi:10.1111/gcb.13153)

5. Tyukavina A, Baccini A, Hansen MC, Potapov P V, Stehman S V, Houghton RA, Krylov AM, Turubaniva S, Goetz SJ. 2015 Aboveground carbon loss in natural and managed tropical forests from 2000 to 2012. *Environ. Res. Lett.* **10**, 1–14.

6. Umunay PM, Gregoire TG, Gopalakrishna T, Ellis PW, Putz FE. 2019 Selective logging emissions and potential emission reductions from reduced-impact logging in the Congo Basin. *For. Ecol. Manage.* **437**, 360–371. (doi:10.1016/j.foreco.2019.01.049)

7. Griscom BW *et al.* 2019 Reduced-impact logging in Borneo to minimize carbon emissions and impacts on sensitive habitats while maintaining timber yields. *For. Ecol. Manage.* **438**, 176–185. (doi:10.1016/j.foreco.2019.02.025)

8. Medjibe VP, Putz FE. 2012 Cost comparisons of reduced-impact and conventional logging in the tropics. *J. For. Econ.* **18**, 242–256. (doi:10.1016/j.jfe.2012.05.001)

9. Brandle JR, Hodges L, Zhou XH. 2004 Windbreaks in North American Agricultural Systems Windbreaks in North American Agricultural Systems. *Agrofor. Syst.* **61**, 65–78.

10. Peri PL, Bloomberg M. 2002 Windbreaks in southern Patagonia, Argentina: A review of research on growth models, windspeed reduction, and effects on crops. *Agrofor. Syst.* **56**, 129–144. (doi:10.1023/A:1021314927209)

11. Cleugh HA *et al.* 2002 The Australian National Windbreaks Program: overview and summary of results. *Aust. J. Exp. Agric.* **42**, 649. (doi:10.1071/EA02003)

12. Tamang B, Andreu MG, Rockwood DL. 2010 Microclimate patterns on the leeside of single-row tree windbreaks during different weather conditions in Florida farms: Implications for improved crop production. *Agrofor. Syst.* **79**, 111–122. (doi:10.1007/s10457-010-9280-4)

13. Baccini A, Walker W, Carvalho L, Farina M, Sulla-Menashe D, Houghton RA. 2017 Tropical forests are a net carbon source based on aboveground measurements of gain and loss. *Science (80-. ).* **358**, 230–234. (doi:10.1126/science.aam5962)

14. Zomer RJ *et al.* 2016 Global Tree Cover and Biomass Carbon on Agricultural Land: The contribution of agroforestry to global and national carbon budgets. *Sci. Rep.* **6**, 29987. (doi:10.1038/srep29987)

15. Ramankutty N, Evan AT, Monfreda C, Foley JA. 2008 Farming the planet: 1. Geographic distribution of global agricultural lands in the year 2000. *Global Biogeochem. Cycles* **22**, 1–19. (doi:10.1029/2007GB002952)

16. Hansen MC *et al.* 2013 High-resolution global maps of 21st-century forest cover change. *Science.* **342**, 850–853. (doi:10.1126/science.1244693)

17. Udawatta, Ranjith P., Jose S. 2011 Carbon Sequestration Potential of Agroforestry Systems. In *Carbon Sequestration Potential of Agroforestry Systems* (eds BM Kumar, PKR Nair), pp. 17–42. Dordrecht: Springer Netherlands.

18. Dinerstein E *et al.* 2017 An Ecoregion-Based Approach to Protecting Half the Terrestrial Realm. *Bioscience* **67**, 534–545. (doi:10.1093/biosci/bix014)

19. Mokany K, Raison RJ, Prokushkin AS. 2006 Critical analysis of root: Shoot ratios in terrestrial biomes. *Glob. Chang. Biol.* **12**, 84–96. (doi:10.1111/j.1365-2486.2005.001043.x)

20. Powers JS, Corre MD, Twine TE, Veldkamp E. 2011 Geographic bias of field observations of soil carbon stocks with tropical land-use changes precludes spatial extrapolation. *Proc. Natl. Acad. Sci. U. S. A.* **108**, 6318–6322. (doi:10.1073/pnas.1016774108)

21. Lassaletta L, Billen G, Garnier J, Bouwman L, Velazquez E, Mueller ND, Gerber JS. 2016 Nitrogen use in the global food system: past trends and future trajectories of agronomic performance, pollution, trade, and dietary demand. *Environ. Res. Lett.* **11**. (doi:10.1088/1748-9326/11/9/095007)

22. Zhang X, Davidson EA, Mauzerall DL, Searchinger TD, Dumas P, Shen Y. 2015 Managing nitrogen for sustainable development. *Nature* **528**, 51–59. (doi:10.1038/nature15743)

23. Lassaletta L, Billen G, Grizzetti B, Anglade J, Garnier J. 2014 50 year trends in nitrogen use efficiency of world cropping systems: The relationship between yield and nitrogen input to cropland. *Environ. Res. Lett.* **9**. (doi:10.1088/1748-9326/9/10/105011)

24. Davidson E a. 2009 The contribution of manure and fertilizer nitrogen to atmospheric nitrous oxide since 1860. *Nat. Geosci.* **2**, 659–662. (doi:10.1038/ngeo608)

25. IPCC. 2013 Working Group I: Contribution to the IPCC Fifth Assessment Report Climate Change 2013: The Physical Science Basis.

26. Bunting P *et al.* 2018 The global mangrove watch - A new 2010 global baseline of mangrove extent. *Remote Sens.* **10**. (doi:10.3390/rs10101669)

27. Worthington T, Spalding M. 2018 Mangrove Restoration Potential: A global map highlighting a critical opportunity. https://doi.org/10.17863/CAM.39153

28. Simard M, Fatoyinbo L, Smetanka C, Rivera-Monroy VH, Castañeda-Moya E, Thomas N, Van der Stocken T. 2019 Mangrove canopy height globally related to precipitation, temperature and cyclone frequency. *Nat. Geosci.* **12**, 40–45.

29. Howard J, Hoyt S, Isensee K, Telszewski M, Pidgeon E, editors. 2014 *Coastal Blue Carbon: Methods for assessing carbon stocks and emissions factors in mangroves, tidal salt marshes, and seagrasses.* Arlington, VA: Conservation International. See https://www.cifor.org/library/5095/.

30. Sanderman J *et al.* 2018 A global map of mangrove forest soil carbon at 30 m spatial resolution. *Environ. Res. Lett.* (doi:10.1088/1748-9326/aabe1c)

31. Giri C, Ochieng E, Tieszen LL, Zhu Z, Singh a., Loveland T, Masek J, Duke N. 2011 Status and distribution of mangrove forests of the world using earth observation satellite data. *Glob. Ecol. Biogeogr.* **20**, 154–159. (doi:10.1111/j.1466-8238.2010.00584.x)

32. Esch T, Heldens W, Hirner A, Keil M, Marconcini M, Roth A, Zeidler J, Dech S, Strano E. 2017 Breaking new ground in mapping human settlements from space – The Global Urban Footprint. *ISPRS J. Photogramm. Remote Sens.* **134**, 30–42. (doi:10.1016/j.isprsjprs.2017.10.012)

33. Murray NJ, Phinn S, DeWitt M, Ferrari R, Johnston R, Lyons MB, Clinton N, Thau D, Fuller R. 2018 The global distribution and trajectory of tidal flats. *Nature* **565**, 222–225.

34. Pekel JF, Cottam A, Gorelick N, Belward AS. 2016 High-resolution mapping of global surface water and its long-term changes. *Nature* **540**, 418–422. (doi:10.1038/nature20584)
