# Supplementary material for: National mitigation potential from natural climate solutions in the tropics
Source: Philos Trans R Soc Lond B Biol Sci. 2020 Jan 27;375(1794):20190126. doi: 10.1098/rstb.2019.0126 (PMC7017762; doi:10.1098/rstb.2019.0126)
Supplement: Supplementary tables [file rstb20190126supp2.docx]

**Supplementary material: Supplemental Tables**

for

**Title:** National mitigation potential from natural climate solutions in the tropics

**Authors:** Bronson W. Griscom, Jonah Busch, Susan C. Cook-Patton, Peter W. Ellis, Jason Funk, Sara M. Leavitt, Guy Lomax, Will Turner, Melissa Chapman, Jens Engelmann, Noel P. Gurwick, Emily Landis, Deborah Lawrence, Yadvinder Malhi, Lisa Schindler Murray, Diego Navarrete, Stephanie Roe, Sabrina Scull, Pete Smith, Charlotte Streck, Wayne S. Walker, Thomas Worthington

**Table S1. Maximum (with safeguards) mitigation potential of 11 natural climate solutions pathways (not including Reforestation).** Units are mean annual
million metric tonnes of CO_2_ equivalents during the period 2030-2050 (TgCO_2_e yr^-1^). Absence of a value indicates that the value is unknown.

|  | **Protect – Forest** | **Protect - Wetland** | **Protect - Wetland** | **Manage - Forest** | **Manage - Forest** | **Manage - Forest** | **Manage - Agriculture** | **Manage - Agriculture** | **Manage - Agriculture** | **Restore - Wetland** | **Restore - Wetland** |
| --- | --- | --- | --- | --- | --- | --- | --- | --- | --- | --- | --- |
| **Country/Territory** | **Avoided Forest Conversion** | **Avoided Peat Impacts** | **Avoided Mangrove Loss** | **Improved Natural Forest Management** | **Reduced Woodfuel Harvest** | **Improved Fire Management (Savannas)** | **Trees in Agricultural Lands** | **Nutrient Management** | **Optimal Grazing Intensity** | **Peat Restoration** | **Mangrove Restoration** |
| Angola | 46.22 | 0.62 | 0.05 | 5.75 | 2.2 | 15.69 | 21.77 | 0 | 1.12 | 2.94 | 0.01 |
| Bangladesh | 2.22 | 0.22 | 0.66 | 0.17 | 4.49 |  | 2.00 | 5.63 | 0.06 | 1.11 | 0.32 |
| Belize | 4.9 | 0.01 | 0.45 | 0.16 | 0.02 |  | 0.13 | 0.03 | 0.02 | 0.03 | 0.15 |
| Benin | 0.71 | 0.03 | 0.00 | 1.66 | 1.01 | 0 | 1.64 | 0 | 0.05 | 0.15 | 0.00 |
| Bolivia | 92.5 | 0.01 |  | 1.53 | 0.41 | 2.56 | 33.11 | 0.01 | 0.89 | 0.04 |  |
| Botswana | 0.01 | 0.06 |  | 0.41 | 0.18 |  | 33.41 | 0.24 | 0.46 | 0.29 |  |
| Brazil | 1072.43 | 1.75 | 3.89 | 4.48 | 25.12 | 9.21 | 340.87 | 10.93 | 10.52 | 8.74 | 1.15 |
| Burkina Faso | 0 | 0.03 |  | 4.59 | 2.04 | 0 | 16.81 | 0 | 0.34 | 0.15 |  |
| Burundi | 0.42 | 0.31 |  | 1.42 | 0.83 | 0.03 | 0.12 | 0 |  | 0.18 |  |
| Cambodia | 53.13 |  | 0.41 | 1.61 | 1.61 |  | 17.83 | 0 | 0.05 |  | 0.13 |
| Cameroon | 20.86 | 0.06 | 0.28 | 16.69 | 2.41 | 0 | 13.87 | 0 | 0.13 | 0.29 | 0.07 |
| Central African Rep. | 16.86 | 0.01 |  | 3.19 | 0.5 | 17.11 | 1.14 | 0 | 0.21 | 0.03 |  |
| Chad | 0.4 |  |  | 2.98 | 1.5 | 1.54 | 114.07 | 0 | 0.93 | 0.02 |  |
| Colombia | 87.74 | 0.1 | 2.50 | 8.79 | 1.8 | 0.64 | 40.27 | 3.56 | 1.84 | 0.09 | 0.51 |
| Costa Rica | 5.84 | 0.01 | 0.12 | 4.74 | 0.56 |  | 1.12 | 0.53 | 0.13 | 0.03 | 0.03 |
| Cote d'Ivoire | 43.65 | 0.47 | 0.04 | 11.35 | 2.95 | 0 | 1.38 | 0 | 0.26 | 0.87 | 0.01 |
| Cuba | 4.76 | 0.56 | 1.82 | 2.2 | 0 |  | 6.99 | 0.23 | 0.21 | 2.07 | 0.38 |
| Dem. Rep. of the Congo (DRC) | 328.63 | 0.94 | 0.20 | 26.97 | 0 | 10.23 | 0.42 | 0 | 0.64 | 1.75 | 0.04 |
| Dominican Republic | 6.48 | 0.01 | 0.08 | 0.2 | 0.9 |  | 2.39 | 0.32 | 0.1 | 0.01 | 0.02 |
| Ecuador | 19.6 | 0.06 | 1.06 | 6.92 | 0.82 |  | 2.35 | 0.83 | 0.38 | 0.29 | 0.25 |
| El Salvador | 1.5 | 0.01 | 0.11 | 2.44 | 0.61 |  | 0.95 | 0.36 | 0.05 | 0.06 | 0.04 |
| Equatorial Guinea | 2.3 |  | 0.08 | 6.88 | 0.07 |  | 0.00 |  |  | 0.01 | 0.02 |
| Eritrea | 0 |  | 0.01 | 0.09 | 0.48 | 0 | 0.00 | 0 | 0.08 |  | 0.00 |
| Ethiopia | 10.46 | 0.38 | 0.00 | 10.38 | 16.53 | 1.09 | 0.04 | 0 | 0.56 | 0.44 |  |
| French Guiana (France) | 2.02 | 0.01 | 0.37 | 0.57 | 0.02 |  | 0.01 |  |  | 0.03 | 0.10 |
| Gabon | 10.62 | 0.07 | 0.39 | 18.24 | 0.18 | 0.13 | 0.27 | 0.01 | 0.04 | 0.06 | 0.09 |
| Gambia, The | 0.01 | 0.01 | 0.11 | 1.16 | 0.18 | 0 | 0.00 | 0 | 0.01 | 0.06 | 0.04 |
| Ghana | 16.4 | 0.01 | 0.12 | 14.5 | 4.16 | 0 | 6.02 | 0 | 0.27 | 0.06 | 0.05 |
| Guatemala | 30.97 | 0.04 | 0.13 | 2.53 | 2.91 |  | 1.67 | 0.76 | 0.33 | 0.04 | 0.05 |
| Guinea | 7.36 | 0.29 | 0.26 | 3.33 | 2.28 | 1.11 | 0.78 | 0 | 0.73 | 1.46 | 0.10 |
| Guinea-Bissau | 1.36 |  | 0.29 | 0.67 | 0.42 | 0.32 | 0.00 |  | 0.11 | 0.02 | 0.10 |
| Guyana | 3.71 | 0.58 | 0.26 | 3.07 | 0.16 | 0 | 1.91 | 0.04 |  | 2.91 | 0.06 |
| Haiti | 0.75 |  | 0.13 |  | 1.19 |  | 1.70 |  | 0.05 |  | 0.03 |
| Honduras | 18.42 | 0.66 | 0.59 | 1.87 | 1.41 |  | 1.59 | 0.41 | 0.29 | 1.09 | 0.17 |
| India | 32.91 | 0.29 | 1.20 | 11.79 | 53.88 | 0 | 175.60 | 89.51 | 0.93 | 1.46 | 0.36 |
| Indonesia | 572.84 | 514.24 | 24.29 | 246.02 | 27.42 | 0.04 | 12.43 | 4.93 | 0.24 | 363.85 | 4.38 |
| Jamaica | 1.27 | 0.04 | 0.06 | 0.59 | 0.3 |  | 0.19 | 0.04 |  | 0.07 | 0.01 |
|  | **Protect - Forest** | **Protect - Wetland** | **Protect - Wetland** | **Manage - Forest** | **Manage - Forest** | **Manage - Forest** | **Manage - Agriculture** | **Manage - Agriculture** | **Manage - Agriculture** | **Restore - Wetland** | **Restore - Wetland** |
| **Country/Territory** | **Avoided Forest Conversion** | **Avoided Peat Impacts** | **Avoided Mangrove Loss** | **Improved Natural Forest Management** | **Reduced Woodfuel Harvest** | **Improved Fire Management (Savannas)** | **Trees in Agricultural Lands** | **Nutrient Management** | **Optimal Grazing Intensity** | **Peat Restoration** | **Mangrove Restoration** |
| Kenya | 12.32 | 3.88 | 0.29 | 0.16 | 6.13 | 0 | 69.24 | 0.26 | 0.64 | 2.91 | 0.08 |
| Laos | 55.8 | 0.06 | 0.00 | 19.61 | 1.02 |  | 1.56 | 0 | 0.05 | 0.29 |  |
| Liberia | 20.5 | 0.08 | 0.03 | 2.56 | 1.1 |  | 0.00 |  | 0.07 | 0.12 | 0.01 |
| Madagascar | 69.7 | 0.94 | 0.61 | 0.87 | 3.15 |  | 30.00 | 0 | 1.78 | 1.78 | 0.19 |
| Malawi | 3.15 | 0.18 | 0.00 | 5.29 | 1.33 | 0.1 | 1.45 | 0.31 | 0.06 | 0.87 |  |
| Malaysia | 171.48 | 57.01 | 2.13 | 19.36 | 0.9 |  | 1.59 | 2.03 |  | 34.93 | 0.39 |
| Mali | 0.05 | 0.03 | 0.00 | 3.2 | 0.86 | 0.32 | 6.84 | 0 | 0.9 | 0.15 |  |
| Mauritania |  | 0.03 | 0.00 | 0.01 | 0.28 |  | 33.35 |  | 0.51 | 0.09 |  |
| Mexico | 64.22 | 0.58 | 7.76 | 18.38 | 4.8 | 0 | 47.48 | 3.95 | 5.23 | 2.91 | 3.41 |
| Mozambique | 56.23 | 0.76 | 1.42 | 7.77 | 3.52 | 3.34 | 9.40 | 0 | 0.79 | 2.33 | 0.61 |
| Myanmar | 65.46 | 0.58 | 3.19 | 25.58 | 6.24 |  | 17.56 | 0 | 0.2 | 2.91 | 1.02 |
| Namibia | 0.03 | 0.01 | 0.00 |  | 0.08 |  | 35.86 | 0.02 | 0.95 | 0.03 |  |
| Nicaragua | 31.71 | 0.06 | 0.79 | 0 | 0.6 |  | 4.69 | 0.22 | 0.37 | 0.29 | 0.24 |
| Niger | 0 | 0.07 |  | 2.75 | 0.96 | 0 | 70.18 | 0 | 0.66 | 0.03 |  |
| Nigeria | 9.72 | 0.71 | 0.97 | 22.71 | 10.07 | 2.77 | 45.38 | 0 | 1.3 | 0.87 | 0.26 |
| Panama | 9.76 | 0.06 | 0.40 | 0.09 | 0.19 |  | 1.27 | 0.1 | 0.09 | 0.29 | 0.08 |
| Papua New Guinea | 26.85 | 27.22 | 1.58 | 19.68 | 1.05 | 0.07 | 0.79 | 0 |  | 14.55 | 0.32 |
| Paraguay | 66.33 | 0.01 | 0.00 | 12.16 | 2.07 | 0.48 | 29.99 | 0 | 1.01 | 0.06 |  |
| Peru | 66.03 | 0.06 | 0.00 | 2.15 | 1.2 |  | 5.53 | 0.96 | 0.86 | 0.29 |  |
| Philippines | 22.04 | 0.05 | 1.72 | 0.34 | 3.29 |  | 7.39 | 0.63 | 0.09 | 0.23 | 0.37 |
| Rep. of Congo | 13.46 | 0.01 | 0.00 | 9.1 | 0.52 |  | 0.23 | 0 | 0.11 | 0.03 |  |
| Rwanda | 0.5 | 0.71 | 0.00 |  | 1.14 | 0.01 | 0.18 | 0 | 0.01 | 0.6 |  |
| Saudi Arabia |  |  | 0.03 |  | 0 |  | 0.24 | 0.82 | 0.15 |  | 0.01 |
| Senegal | 0.04 | 0.02 | 0.55 | 4.17 | 1.39 | 0 | 0.00 | 0.03 | 0.14 | 0.02 | 0.18 |
| Sierra Leone | 8.71 | 0.08 | 0.08 | 0.72 | 0.89 | 0.48 | 0.00 |  | 0.33 | 0.12 | 0.03 |
| Solomon Islands | 2.49 |  | 0.18 | 10.47 | 0.02 |  | 0.00 |  |  | 0.01 | 0.04 |
| Somalia | 0.07 | 0.03 | 0.01 | 0.43 | 1.88 | 0 | 107.32 |  | 0.24 | 0.15 | 0.00 |
| South Sudan | 2.38 |  |  |  | 0 |  | 37.20 |  | 1.06 |  |  |
| Sri Lanka | 2.64 |  | 0.24 | 2.53 | 1.88 |  | 1.82 | 1.22 | 0.04 | 0.06 | 0.07 |
| Sudan | 0.02 | 0.58 | 0.00 | 4.53 | 4.74 | 0 | 234.92 | 0.48 | 1.04 | 2.91 | 0.00 |
| Suriname | 3.22 | 0.06 | 0.68 | 3.85 | 0.03 |  | 0.15 | 0.04 |  | 0.29 | 0.16 |
| Tanzania | 41.44 | 0.11 | 0.31 | 6.22 | 8.94 | 6.03 | 32.81 | 0 | 0.95 | 0.26 | 0.08 |
| Thailand | 45.14 | 0.25 | 1.37 |  | 5.67 |  | 32.80 | 3.63 |  | 1.57 | 0.41 |
| Timor-Leste | 0.7 |  | 0.00 |  | 0.02 |  | 0.46 |  | 0.01 |  | 0.00 |
| Togo | 0.63 | 0.07 |  | 0.43 | 1.02 | 0 | 1.08 | 0 | 0.05 | 0.06 |  |
| Trinidad &Tobago | 0.57 | 0.01 | 0.04 | 0.23 | 0.01 |  | 0.06 |  |  | 0.01 | 0.01 |
| Uganda | 14.07 | 8.8 | 0.00 | 23.12 | 6.37 | 0.24 | 2.35 | 0 | 0.31 | 14.55 |  |
| Venezuela | 34 | 1.11 | 1.09 | 2.41 | 0.67 | 0.29 | 29.01 | 1.86 | 0.94 | 2.62 | 0.28 |
| Vietnam | 54.59 | 0.76 | 1.13 | 15.53 | 6.76 |  | 9.63 | 2.95 | 0.21 | 3.81 | 0.41 |
| Yemen |  |  | 0.00 |  | 0 |  | 6.77 | 0 | 0.1 |  | 0.00 |
| Zambia | 31.8 | 1.88 | 0.00 | 2.51 | 3.11 | 14.49 | 10.36 | 0.56 | 1.11 | 3.49 |  |
| Zimbabwe | 4.87 | 0.44 | 0.00 | 1.47 | 2.93 | 0.81 | 25.36 |  | 1.08 | 0.73 |  |

**Table S2. Maximum climate mitigation potential of avoided mangrove loss and mangrove restoration in countries and territories not included in Table S1 or S3**, due either to small size (<10,000 km^2^) or countries with majority land mass outside of the tropical latitudes. Units are in metric tonnes of CO₂ per year (MgCO₂e yr^-1^).

| **Country/Territory** | **Avoided Mangrove Loss** | **Mangrove Restoration** |
| --- | --- | --- |
| Australia | 2,700,028 | 789,309 |
| Bahamas | 993,818 | 166,951 |
| Bahrain | 16 | 3 |
| Brunei | 78,262 | 16,412 |
| China | 62,376 | 15,043 |
| China - Hong Kong | 957 | 354 |
| Djibouti | 281 | 113 |
| Dominica | 4 | 1 |
| Egypt | 446 | 116 |
| Fiji | 60,441 | 13,665 |
| France - Guadeloupe | 1,689 | 398 |
| France - Martinique | 131 | 30 |
| France - Mayotte | 298 | 65 |
| France - Saint-Martin | 180 | 68 |
| Grenada | 195 | 56 |
| Iran | 12,521 | 3,694 |
| Japan | 2,449 | 341 |
| Mauritius | 1,778 | 601 |
| Micronesia | 1,641 | 301 |
| Netherlands - Aruba | 1,303 | 454 |
| Netherlands - Bonaire | 5,445 | 647 |
| Netherlands - Curaçao | 1,477 | 491 |
| New Caledonia | 48,264 | 15,953 |
| New Zealand | 142,912 | 34,047 |
| Oman | 452 | 190 |
| Pakistan | 377,207 | 78,936 |
| Palau | 2,370 | 268 |
| Qatar | 1,170 | 164 |
| Saint Kitts and Nevis | 100 | 4 |
| Saint Lucia | 47 | 12 |
| Saint Vincent and the Grenadines | 45 | 8 |
| **Country/Territory** | **Avoided Mangrove Loss** | **Mangrove Restoration** |
| Samoa | 5 | 7 |
| Seychelles | 361 | 67 |
| Singapore | 113 | 24 |
| South Africa | 9,838 | 3,571 |
| Taiwan (Republic of China) | 2,384 | 581 |
| Tonga | 225 | 710 |
| United Arab Emirates | 37,518 | 11,038 |
| United Kingdom - Anguilla | 77 | 1 |
| United Kingdom - Antigua and Barbuda | 1,356 | 332 |
| United Kingdom - British Virgin Islands | 44 | 10 |
| United Kingdom - Cayman Islands | 21,289 | 3,386 |
| United Kingdom - Turks and Caicos | 95,854 | 9,862 |
| United States - Puerto Rico | 32,295 | 8,749 |
| United States - Virgin Islands | 300 | 88 |
| United States (except Alaska,  Virgin Islands, Puerto Rico) | 2,031,055 | 532,420 |
| Vanuatu | 1185 | 232 |

**Table S3. Mitigation potential of 12 natural climate solutions pathways at “cost-effective” levels (<100 USD MgCO_2_e^-1^).** Units are mean annual million metric tonnes of CO_2_ equivalents during the period 2030-2050 (TgCO_2_e yr^-1^). Absence of a value indicates that the value is unknown.

|  | **Protect - Forest** | **Protect - Wetland** | **Protect - Wetland** | **Manage - Forest** | **Manage - Forest** | **Manage - Forest** | **Manage - Agriculture** | **Manage - Agriculture** | **Manage - Agriculture** | **Restore - Forest** | **Restore - Wetland** | **Restore - Wetland** |
| --- | --- | --- | --- | --- | --- | --- | --- | --- | --- | --- | --- | --- |
| **Country/Territory** | **Avoided Forest Conversion** | **Avoided Peat Impacts** | **Avoided Mangrove Loss** | **Improved Natural Forest Management** | **Reduced Woodfuel Harvest** | **Improved Fire Management (Savannas)** | **Trees in Agricultural Lands** | **Nutrient Management** | **Optimal Grazing Intensity** | **Reforestation** | **Peat Restoration** | **Mangrove Restoration** |
| Angola | 36.98 | 0.56 | 0.04 | 4.38 | 0.66 | 4.71 | 12.06 | 0.00 | 0.67 | 38.45 | 1.41 | 0.00 |
| Bangladesh | 1.78 | 0.20 | 0.59 | 0.13 | 1.35 | 0.00 | 0.75 | 5.07 | 0.04 | 3.05 | 0.53 | 0.10 |
| Belize | 3.92 | 0.01 | 0.41 | 0.12 | 0.01 | 0.00 | 0.05 | 0.03 | 0.01 | 0.60 | 0.01 | 0.05 |
| Benin | 0.57 | 0.03 | 0.00 | 1.20 | 0.30 |  | 0.62 | 0.00 | 0.03 | 2.65 | 0.07 | 0.00 |
| Bolivia | 74.00 | 0.01 | 0.00 | 1.11 | 0.12 | 0.77 | 19.10 | 0.01 | 0.53 | 24.60 | 0.02 | 0.00 |
| Botswana | 0.01 | 0.05 | 0.00 | 0.30 | 0.05 | 0.00 | 19.78 | 0.22 | 0.28 | 2.11 | 0.14 | 0.00 |
| Brazil | 857.94 | 1.58 | 3.50 | 3.41 | 7.54 | 2.76 | 166.85 | 9.83 | 6.31 | 287.56 | 4.20 | 0.35 |
| Burkina Faso | 0.00 | 0.03 | 0.00 | 3.32 | 0.61 |  | 7.06 | 0.00 | 0.20 | 0.36 | 0.07 | 0.00 |
| Burundi | 0.34 | 0.28 | 0.00 | 1.03 | 0.25 | 0.01 | 0.05 | 0.00 | 0.00 | 1.94 | 0.09 | 0.00 |
| Cambodia | 42.50 | 0.00 | 0.37 | 1.23 | 0.48 | 0.00 | 8.99 | 0.00 | 0.03 | 11.23 | 0.00 | 0.04 |
| Cameroon | 16.69 | 0.05 | 0.25 | 12.77 | 0.72 |  | 7.73 | 0.00 | 0.08 | 12.29 | 0.14 | 0.02 |
| Central African Rep | 13.49 | 0.01 | 0.00 | 2.44 | 0.15 | 5.13 | 0.61 | 0.00 | 0.13 | 13.89 | 0.01 | 0.00 |
| Chad | 0.32 | 0.00 | 0.00 | 2.16 | 0.45 | 0.46 | 66.39 | 0.00 | 0.56 | 2.34 | 0.01 | 0.00 |
| Colombia | 70.19 | 0.09 | 2.25 | 6.38 | 0.54 | 0.19 | 21.85 | 3.21 | 1.10 | 37.99 | 0.04 | 0.15 |
| Costa Rica | 4.67 | 0.01 | 0.11 | 3.45 | 0.17 | 0.00 | 0.51 | 0.48 | 0.08 | 2.66 | 0.01 | 0.01 |
| Cote d'Ivoire | 34.92 | 0.42 | 0.04 | 8.68 | 0.89 | 0.00 | 0.75 | 0.00 | 0.16 | 16.72 | 0.42 | 0.00 |
| Cuba | 3.81 | 0.50 | 1.64 | 1.59 | 0.00 | 0.00 | 2.80 | 0.21 | 0.13 | 4.84 | 0.99 | 0.11 |
| Dem Rep of Congo | 262.90 | 0.85 | 0.18 | 21.10 | 0.00 | 3.07 | 0.19 | 0.00 | 0.38 | 50.95 | 0.84 | 0.01 |
| Dominican Republic | 5.18 | 0.01 | 0.07 | 0.14 | 0.27 | 0.00 | 1.11 | 0.29 | 0.06 | 2.29 | 0.00 | 0.01 |
| Ecuador | 15.68 | 0.05 | 0.96 | 5.03 | 0.25 | 0.00 | 1.07 | 0.74 | 0.23 | 8.85 | 0.14 | 0.08 |
| El Salvador | 1.20 | 0.01 | 0.10 | 1.77 | 0.18 | 0.00 | 0.39 | 0.32 | 0.03 | 1.34 | 0.03 | 0.01 |
| Equatorial Guinea | 1.84 | 0.00 | 0.07 | 5.68 | 0.02 | 0.00 | 0.00 | 0.00 | 0.00 | 0.24 | 0.00 | 0.01 |
| Eritrea | 0.00 | 0.00 | 0.01 | 0.06 | 0.14 | 0.00 | 0.00 | 0.00 | 0.05 | 0.03 | 0.00 | 0.00 |
| Ethiopia | 8.37 | 0.34 | 0.00 | 7.52 | 4.96 | 0.33 | 0.02 | 0.00 | 0.34 | 24.11 | 0.21 | 0.00 |
| French Guiana (France) | 1.62 |  | 0.34 | 0.45 |  |  | 0.00 |  |  |  |  | 0.03 |
| Gabon | 8.50 | 0.06 | 0.35 | 15.05 | 0.05 | 0.04 | 0.14 | 0.01 | 0.02 | 3.21 | 0.03 | 0.03 |
| Gambia, The | 0.01 | 0.01 | 0.10 | 0.89 | 0.05 |  | 0.00 | 0.00 | 0.01 | 0.09 | 0.03 | 0.01 |
| Ghana | 13.12 | 0.01 | 0.10 | 11.34 | 1.25 |  | 2.69 | 0.00 | 0.16 | 8.24 | 0.03 | 0.02 |
| Guatemala | 24.78 | 0.04 | 0.12 | 1.83 | 0.87 | 0.00 | 0.67 | 0.69 | 0.20 | 5.49 | 0.02 | 0.01 |
| Guinea | 5.89 | 0.26 | 0.23 | 2.55 | 0.68 | 0.33 | 0.40 | 0.00 | 0.44 | 10.76 | 0.70 | 0.03 |
| Guinea-Bissau | 1.09 | 0.00 | 0.26 | 0.52 | 0.13 | 0.10 | 0.00 | 0.00 | 0.07 | 1.02 | 0.01 | 0.03 |
| Guyana | 2.97 | 0.52 | 0.24 | 2.45 | 0.05 |  | 0.95 | 0.03 | 0.00 | 3.02 | 1.40 | 0.02 |
| Haiti | 0.60 | 0.00 | 0.12 | 0.00 | 0.36 | 0.00 | 0.65 | 0.00 | 0.03 | 1.31 | 0.00 | 0.01 |
| Honduras | 14.74 | 0.59 | 0.53 | 1.36 | 0.42 | 0.00 | 0.61 | 0.36 | 0.17 | 4.59 | 0.52 | 0.05 |
| India | 26.33 | 0.26 | 1.08 | 8.54 | 16.16 | 0.00 | 66.21 | 80.56 | 0.56 | 52.11 | 0.70 | 0.11 |
| Indonesia | 458.27 | 462.82 | 21.86 | 187.49 | 8.23 | 0.01 | 4.83 | 4.44 | 0.14 | 66.68 | 174.65 | 1.31 |
| Jamaica | 1.02 | 0.04 | 0.05 | 0.43 | 0.09 | 0.00 | 0.08 | 0.04 | 0.00 | 0.48 | 0.03 | 0.00 |
|  | **Protect - Forest** | **Protect - Wetland** | **Protect - Wetland** | **Manage - Forest** | **Manage - Forest** | **Manage - Forest** | **Manage - Agriculture** | **Manage - Agriculture** | **Manage - Agriculture** | **Manage - Agriculture** | **Restore - Wetland** | **Restore - Wetland** |
| **Country/Territory** | **Avoided Forest Conversion** | **Avoided Peat Impacts** | **Avoided Mangrove Loss** | **Improved Natural Forest Management** | **Reduced Woodfuel Harvest** | **Improved Fire Management (Savannas)** | **Trees in Agricultural Lands** | **Nutrient Management** | **Optimal Grazing Intensity** | **Reforestation** | **Peat Restoration** | **Mangrove Restoration** |
| Kenya | 9.86 | 3.49 | 0.26 | 0.12 | 1.84 |  | 40.26 | 0.23 | 0.38 | 10.26 | 1.40 | 0.02 |
| Laos | 44.64 | 0.05 | 0.00 | 14.95 | 0.31 | 0.00 | 0.58 | 0.00 | 0.03 | 10.02 | 0.14 | 0.00 |
| Liberia | 16.40 | 0.07 | 0.03 | 1.96 | 0.33 | 0.00 | 0.00 | 0.00 | 0.04 | 1.29 | 0.06 | 0.00 |
| Madagascar | 55.76 | 0.85 | 0.55 | 0.67 | 0.95 | 0.00 | 17.35 | 0.00 | 1.07 | 21.35 | 0.85 | 0.06 |
| Malawi | 2.52 | 0.16 | 0.00 | 4.05 | 0.40 | 0.03 | 0.59 | 0.28 | 0.04 | 3.17 | 0.42 | 0.00 |
| Malaysia | 137.18 | 51.31 | 1.91 | 14.76 | 0.27 | 0.00 | 0.65 | 1.82 | 0.00 | 10.05 | 16.77 | 0.12 |
| Mali | 0.04 | 0.03 | 0.00 | 2.32 | 0.26 | 0.10 | 3.76 | 0.00 | 0.54 | 0.79 | 0.07 | 0.00 |
| Mauritania | 0.00 | 0.03 | 0.00 | 0.01 | 0.08 | 0.00 | 19.53 | 0.00 | 0.31 | 0.01 | 0.04 | 0.00 |
| Mexico | 51.38 | 0.52 | 6.98 | 13.31 | 1.44 | 0.00 | 23.87 | 3.55 | 3.14 | 46.18 | 1.40 | 1.02 |
| Mozambique | 44.98 | 0.68 | 1.28 | 5.63 | 1.06 | 1.00 | 5.10 | 0.00 | 0.47 | 33.34 | 1.12 | 0.18 |
| Myanmar | 52.37 | 0.52 | 2.87 | 19.49 | 1.87 | 0.00 | 6.63 | 0.00 | 0.12 | 29.19 | 1.40 | 0.31 |
| Namibia | 0.02 | 0.01 | 0.00 | 0.00 | 0.02 | 0.00 | 21.41 | 0.02 | 0.57 | 0.67 | 0.01 | 0.00 |
| Nicaragua | 25.37 | 0.05 | 0.71 | 0.00 | 0.18 | 0.00 | 2.39 | 0.20 | 0.22 | 5.77 | 0.14 | 0.07 |
| Niger | 0.00 | 0.06 | 0.00 | 1.99 | 0.29 | 0.00 | 37.84 | 0.00 | 0.40 | 0.03 | 0.01 | 0.00 |
| Nigeria | 7.78 | 0.64 | 0.87 | 16.45 | 3.02 | 0.83 | 20.06 | 0.00 | 0.78 | 20.76 | 0.42 | 0.08 |
| Panama | 7.81 | 0.05 | 0.36 | 0.07 | 0.06 | 0.00 | 0.48 | 0.09 | 0.05 | 2.82 | 0.14 | 0.02 |
| Papua New Guinea | 21.48 | 24.50 | 1.42 | 15.00 | 0.32 | 0.02 | 0.29 | 0.00 | 0.00 | 10.89 | 6.98 | 0.10 |
| Paraguay | 53.06 | 0.01 | 0.00 | 8.83 | 0.62 | 0.14 | 16.78 | 0.00 | 0.61 | 15.77 | 0.03 | 0.00 |
| Peru | 52.82 | 0.05 | 0.00 | 1.56 | 0.36 | 0.00 | 2.94 | 0.87 | 0.52 | 19.75 | 0.14 | 0.00 |
| Philippines | 17.63 | 0.05 | 1.55 | 0.25 | 0.99 | 0.00 | 2.77 | 0.57 | 0.05 | 17.67 | 0.11 | 0.11 |
| Republic of Congo | 10.77 | 0.01 | 0.00 | 6.96 | 0.16 | 0.14 | 0.11 | 0.00 | 0.07 | 8.81 | 0.01 | 0.00 |
| Rwanda | 0.40 | 0.64 | 0.00 | 0.00 | 0.34 | 0.00 | 0.08 | 0.00 | 0.01 | 1.98 | 0.29 | 0.00 |
| Saudi Arabia | 0.00 | 0.00 | 0.03 | 0.00 | 0.00 | 0.00 | 0.09 | 0.74 | 0.09 | 0.00 | 0.00 | 0.00 |
| Senegal | 0.03 | 0.02 | 0.50 | 3.19 | 0.42 |  | 0.00 | 0.02 | 0.08 | 0.74 | 0.01 | 0.06 |
| Sierra Leone | 6.97 | 0.07 | 0.07 | 0.57 | 0.27 | 0.14 | 0.00 | 0.00 | 0.20 | 3.29 | 0.06 | 0.01 |
| Solomon Islands | 1.99 | 0.00 | 0.16 | 7.98 | 0.01 | 0.00 | 0.00 | 0.00 | 0.00 | 0.06 | 0.00 | 0.01 |
| Somalia | 0.06 | 0.03 | 0.01 | 0.31 | 0.56 | 0.00 | 63.30 | 0.00 | 0.14 | 4.56 | 0.07 | 0.00 |
| South Sudan | 1.90 | 0.00 | 0.00 | 0.00 | 0.00 | 0.00 | 20.49 | 0.00 | 0.64 | 21.56 | 0.00 | 0.00 |
| Sri Lanka | 2.11 | 0.00 | 0.22 | 1.93 | 0.56 | 0.00 | 0.68 | 1.09 | 0.02 | 4.23 | 0.03 | 0.02 |
| Sudan | 0.02 | 0.52 | 0.00 | 3.28 | 1.42 |  | 134.87 | 0.44 | 0.62 | 0.63 | 1.40 | 0.00 |
| Suriname | 2.58 | 0.05 | 0.61 | 3.07 | 0.01 | 0.00 | 0.06 | 0.03 | 0.00 | 1.44 | 0.14 | 0.05 |
| Tanzania | 33.15 | 0.10 | 0.28 | 4.51 | 2.68 | 1.81 | 17.50 | 0.00 | 0.57 | 36.16 | 0.12 | 0.03 |
| Thailand | 36.11 | 0.23 | 1.23 | 0.00 | 1.70 | 0.00 | 12.29 | 3.27 | 0.00 | 29.35 | 0.75 | 0.12 |
| Timor-Leste | 0.56 | 0.00 | 0.00 | 0.00 | 0.01 | 0.00 | 0.17 | 0.00 | 0.01 | 0.77 | 0.00 | 0.00 |
| Togo | 0.50 | 0.06 | 0.00 | 0.33 | 0.31 |  | 0.44 | 0.00 | 0.03 | 2.04 | 0.03 | 0.00 |
| Trinidad & Tobago | 0.46 | 0.01 | 0.03 | 0.17 | 0.00 | 0.00 | 0.03 | 0.00 | 0.00 | 0.20 | 0.00 | 0.00 |
| Uganda | 11.26 | 7.92 | 0.00 | 18.08 | 1.91 | 0.07 | 1.09 | 0.00 | 0.19 | 8.21 | 6.98 | 0.00 |
| Venezuela | 27.20 | 1.00 | 0.99 | 1.75 | 0.20 | 0.09 | 13.99 | 1.68 | 0.56 | 23.62 | 1.26 | 0.08 |
| Vietnam | 43.67 | 0.68 | 1.02 | 11.83 | 2.03 | 0.00 | 3.64 | 2.65 | 0.13 | 18.41 | 1.83 | 0.12 |
| Yemen | 0.00 | 0.00 | 0.00 | 0.00 | 0.00 | 0.00 | 3.97 | 0.00 | 0.06 | 0.06 | 0.00 | 0.00 |
| Zambia | 25.44 | 1.69 | 0.00 | 1.82 | 0.93 | 4.35 | 5.28 | 0.50 | 0.67 | 25.80 | 1.68 | 0.00 |
| Zimbabwe | 3.90 | 0.40 | 0.00 | 1.07 | 0.88 | 0.24 | 13.50 | 0.00 | 0.65 | 7.25 | 0.35 | 0.00 |
